# Wanted—A State Examination

**Published:** 1907-11-09

**Authors:** 


### WANTED—A STATE EXAMINATION.

Dr. Allchin's address on "Some Problems of Medical Education in London," delivered at the opening of the winter session of King's College Medical School, deserves the closest attention of those who are interested in medical education in this country. As teacher and examiner Dr. Allchin is fully qualified to speak on the subject with which he dealt; as one who has made a close study of the methods of education in this country his views and suggestions are worthy of earnest consideration. No one reading his carefully-thought-out and moderately-phrased exposition of the shortcomings, the defects, and the anomalies of the system which prevails at present, can fail to be struck with the fact that, to a greater extent in London perhaps than elsewhere, there is need of reform both in medical examinations and in the curriculum as a whole. His indictment of the university examinations is doubtless open to criticism. "The examinational position of a student constantly belies his performance in after life, and men receive appointments on the strength of examinational distinctions that their subsequent work fails to justify. It is by no means those who come out on the top of the lists who make the most successful practitioners, or who afterwards contribute most to the advancement of special branches of our science, or who become the most capable teachers. . . . Much of the loose talk and writing that one hears and sees on this subject would seem to suggest that the end and object of medical training was to pass a degree of a high standard." As an examiner he affirms, what will not be news to most of us, that the stringency of university examinations is scarcely so real or the test so far-reaching as is commonly imagined, and that the examinations of the Conjoint Board "are, taken as a whole, as satisfactory as any, and more so than many."

We do not wish, nor do we intend, to criticise these dicta. The whole system of medical education, with the intricacies of university systems, the anomalies of degree tests, and the belittling of qualifications as opposed to graduate honours, is one which few will attempt to defend as it stands. Most, indeed, will agree with Dr. Allchin that it is a reproach to the organisation of medical teaching. But criticism, to do good, should do more than merely destroy. It should offer a hint as to betterment, improvement, reorganisation, and amendment, and those interested in the subject will naturally turn to Dr. Allchin's recommendations for altering matters so as to establish some degree of uniformity. And it is interesting to note that he expresses himself strongly in favour of "the often advocated State Examination, carried out by examiners appointed and paid by the Government." This, the ideal plan as it would seem, is working advantageously in other countries, where the mere attainment of a university degree confers no right to practise unless the holder has satisfied the State of his qualification for his profession by passing the State examination. The arguments in favour of establishing such a uniform system in England are many, and they appeal forcibly to every lover of order and system. It would do away with the vexed question of the title of doctor; it would introduce a uniform system, which would contribute materially to raising the standard of medical education generally; it would involve the virtual abolition of several of the present licensing bodies; and it would leave the universities and the colleges free to fix a higher qualification for such as desire to specialise in some form or other. On the other side there appears to be no argument of vital importance why such a system should not be initiated. Many of the difficulties raised by supporters of the present system are difficulties of detail, and can easily be smoothed over by combined action on the part of the several licensing and examining bodies. The abortive attempt in 1879 to establish a single portal to the profession was a discouragement, but there exists no reason why some attempt should not be made at present to reopen the question with a hope of obtaining legislative sanction for the plans then put forward. The creation of a new Royal Commission to report on the whole subject of medical education would be hailed by most teachers and examiners with undisguised satisfaction, and would lead to modifications and improvements which will tend towards uniformity. Nothing short of a thorough revision of existing schemes of medical education will receive universal approbation. The time for compromises such as those which led to the establishment of the present Conjoint diploma examinations is past. The means of entry into the medical profession are so numerous, in many ways so anomalous, so unequal, that the time has come for the State to take action in the matter, to introduce uniformity, and to sweep aside, by the creation of a State examination that shall be an efficient test of the merits of individual candidates, the fictitious distinctions between degrees and qualifications, and the invidious usurpation of preeminence by separate schools and distinct universities, which at present embarrass the student and confuse the public.

